# Virtual chemical inversion - a novel fat suppression technique for T1-weighted cardiac imaging with improved delineation of the fat-myocardium interface

**DOI:** 10.1186/1532-429X-15-S1-P19

**Published:** 2013-01-30

**Authors:** Elizabeth Jenista, David Wendell, Han W Kim, Stephen Darty, Anna Lisa Crowley, Enn-Ling Chen, Wolfgang G Rehwald, Michele Parker, Raymond J Kim

**Affiliations:** 1 Duke University, Durham, NC, USA; 2 Siemens Healthcare, Chicago, IL, USA

## Background

Fat-saturation and T1-weighting are fundamental for tissue differentiation, but combining these techniques is challenging. For instance, standard chemically selective saturation (CHESS) pulses provide insufficient fat-suppression with routine delayed enhancement imaging [1]. Fat-water separation using multi-echo Dixon techniques can robustly separate fat and water into individual images, but in the fat-suppressed (water only) image, fat may retain a significant amount of signal making identification of the fat-water border difficult.

We previously described a new technique (Virtual Chemical Inversion, VCI, [1]) which has the advantage of providing T1-weighted images with and without fat suppression in a single acquisition without the need for additional data acquisition, RF pulses, or increased scan time. In this study, we compare the ability of the CHESS, a multi-echo Dixon technique (VARPRO, [2]), and our new method (VCI) to completely delineate the myocardium-fat interface in patients, and the level of fat suppression in phantoms.

## Methods

**Phantoms** - A phantom with tubes of oil and water was imaged at 1.5T (MAGNETOM Avanto) using CHESS, VARPRO and VCI. B0 offsets ranging from 0 Hz to 100 Hz were tested. Regions of interest (ROIs) were drawn in the fat and water tubes and fat suppression was evaluated by calculating the fat-water signal ratio.

**Patients** - Patient images were acquired in 10 consecutive patients at 1.5T with VARPRO, VCI, and DIR-TSE with SPAIR, a commonly used method for pre-contrast fat suppression [3]. Two experienced readers estimated the percentage of the visible RV myocardium - fat interface. ROIs were drawn in the myocardium and pericardial fat. The ratio of fat to myocardium signal was calculated. Analyses were performed using ANOVA with Bonferroni correction.

## Results

**Figure 1:**
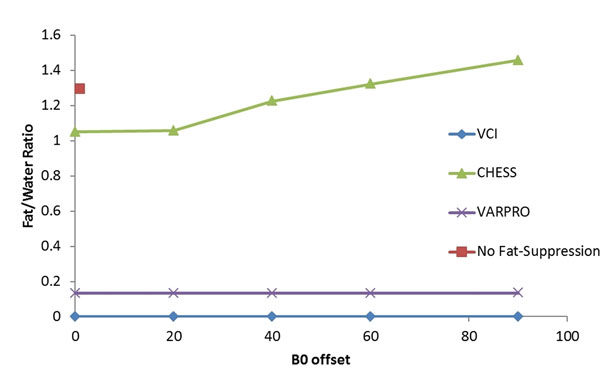
Phantom results. In the phantom, VCI provided uniform fat signal suppression over a range of 90 Hz. The VARPRO provided slightly less fat suppression, but was uniform over the entire range of offsets. The CHESS provided no significant fat suppression for any offset.

**Figure 2:**
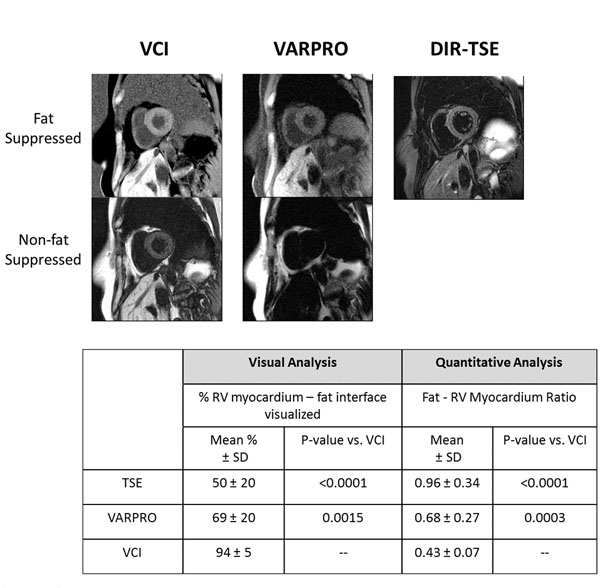
Patient results. Top: Patient example images comparing VCI, VARPRO and DIR-TSE. Fat suppression in the DIR-TSE is done using a SPAIR pulse (Spectrally Attenuated Inversion Recovery). The VCI provides clear contrast between the RV-myocardium and pericardial fat, while the entire interface is not clearly delineated on the VARPRO and DIR-TSE fat suppressed images. Bottom - Comparison of the visual and quantitative scores in patients. The VCI allows an average of 94% of the interface to be seen, and has the lowest fat-myocardium ratio. Note that smaller fat-myocardium ratios signify improved fat suppression.

In the phantom, VCI and VARPRO led to significant fat signal reduction, with no variation in the fat-water signal over a range of 90 Hz. The CHESS method provided little fat suppression for clinical readout times, and was sensitive to resonance offset (Figure 1). In patients (Figure 2), on average, 94% of the RV myocardium-fat interface was seen with VCI, which was statistically different from both VARPRO(69%) and DIR-TSE (50%). By quantitative analysis, VCI was shown to have the lowest fat-myocardium ratio(0.43) followed by VARPRO (0.86) and DIR-TSE (0.96)(Figure 2).

## Conclusions

VCI exhibited excellent fat-water separation in both phantoms and patients, consistently providing fat suppression to a higher degree than both DIR-TSE and VARPRO, allowing for nearly complete delineation of the RV myocardium- fat interface.

## Funding

None
